# On the Mode of Action of Alcohol in the Treatment of Disease

**Published:** 1861

**Authors:** Edward Smith

**Affiliations:** Assistant-Physician to the Hospital for Consumption and Diseases of the Chest Brompton, London


### ON THE MODE OF ACTION OF ALCOHOL IN THE TREATMENT OF DISEASE.

BY EDWARD SMITH, M. D., L. L. B., F. R. S.

*Assistant-Physician to the Hospital for Consumption and Diseases of the Chest Brompton, London.*

The subject to which I propose to direct attention is one which for some time past has excited an unusual amount of professional interest, and has led to some angry discussion. Its importance cannot well be exaggerated, since in the quantities which alcohol has of late years been administered in diseases, this agent must be powerful for good or evil; and the more so now that the plan, having passed out of the hands of him who introduced it, may not unlikely be adopted by many of his pupils and admirers, who have not so large an experience or so well informed a sense of discrimination. But, whilst impressed with these facts, my object now is not principally to keep up the present controversy, but, since additional information on the subject has been recently obtained, to discuss its merits on scientific grounds, and to remove it further from the domain of hypothesis and empiricism. I wish to show what is the true action of alcohol, and thence the general conditions of system to which it is fitted, rather than to discuss minutely the specific diseases in which it may be employed. The same conditions exist in some stages of many diseases, and hence it is better to confine our attention to the separate indications for its use, than to regard the whole phenomena of a disease as a unity, to be treated on a uniform plan. The indications being given, it will be easy to specify the diseases and stages of diseases to which its action is suitable.

In adducing the proofs of the action of alcohol in diseases to which it is suitable, I presume we are entitled to cite its action in health; for whilst the *degree* of action in the former may be different from that in the latter, the *direction* of its action must be the same in both. The indications for its use in disease can never be opposed to its indications in health, but tolerance of its action may be very different in the two conditions. This is an important consideration; for whilst we have much proof of its action in health, we have very little, indeed, in disease. At present we only seek for its mode, and not its degree of action.

The knowledge of its action in health may be sought for from two sources; that which is readily cognisant to the senses, and that from scientific research. The former source of information is perhaps more valuable in this than in any similar inquiry, for the universal employment of alcohols and their decided effects render them objects of popular observation.

*The Brain.*—Alcohols, in moderate quantity, are popularly known to excite the spirits, and to quicken certain qualities of the mind, but to lessen the power of concentration and the clearness of thought; and, when taken in larger quantities, to disturb the harmony of the mental actions so as to render the person furious and ungovernable, to lesson the power of perception and sensibility, and ultimately to produce coma.

*The Skin and Heart.*—They also make the hands and face red, hot, and swollen, and increase the force of the heart's action. The skin is commonly dry as well as hot.

*Heat.*—They yield a pleasant warmth to the stomach and alimentary canal, and the more so when the skin is cold.

*The Muscles.*—The muscular force seems at first sometimes to be increased, for a man in his fury can exert unwonted strength; but this is only in appearance, and commonly there is less disposition to move, and less power of co-ordinating the muscles; whilst relaxation of the features exists, and ultimately there is inability to move.

*The Kidneys.*—In some persons there is diuresis, whether from increased secretion of urine or from lessened power of the sphincter.

*The Lungs.*—The breathing in approaching coma is feeble, labored or irregular.

These effects are temporary, enduring two or three hours; and after the excitement there are, in very varying degrees, depression of the spirits and general lassitude, mental prostration, dry furred tongue, unpleasant taste, headache, derangement of digestion, and loss of appetite. Soda-water and brandy in the morning are as refreshing to some persons after as the wine or spirit was pleasant during the debauch.

Persons vary very much as to the amount of alcohol which they can take, and as to its effect upon the skin and kidneys; but, as a rule, one who perspires and passes urine freely can take the larger quantity. A degree of training is also necessary, so that persons become accustomed to the use of alcohol—that is to say, they gain some power of adaptation of the system to its presence. Alcohol-drinkers in excess are known to be unhealthy, and not equal to others in mental or physical power. They also suffer from loss of appetite, deficient assimilation, and local effects on the brain, stomach, liver and kidneys. Ale-drinkers are more subject to inflammation than spirit-drinkers. Such are popular observations.

In a prolonged inquiry upon myself and another, we took the alcohols in moderate quantity, duly diluted, on an empty stomach, in the morning and during rest; and we also noticed most carefully the general effects, and the moment of their occurrence. The parts of the system influenced by the alcohols, and the order of the occurrence of their symptoms, were as follows:

1. Upon the heart, and doubtless from the local action of the alcohol.

2. Upon the brain, as shown by the consciousness, mental and sensual perceptions.

3. Upon the cerebro-spinal tract, as shown by the muscular system, and upon the reflex function of the spinal cord.

4. Upon the respiratory tract.

5. Upon the sympathetic system; but as it is not possible to show the moment when the secretion of urine, for example, is interferred with, it may be that this should have had an earlier place in this order of sequence.

The details of these actions and the period of their occurrence may be thus epitomized, it being borne in mind that the experiments were made in the morning during fasting:

(*a*) In from two to eight minutes a sensation of fullness at the crown and the back part of the head, or at the temples, according to the kind of spirit taken. This was due, doubtless, in great part, to the increased force of the heart.

(*b*) In from two to seven minutes the mind was disturbed. Consciousness, the power of fixing the attention, the perception of light, and we believe of sound also, was lessened; the power of directing and co-ordinating the muscles was also lessened, while there was a very marked, peculiar, continuous, purring or thrilling, and not unpleasant sensation, passing from above downwards through the whole system. This latter symptom was the most pronounced in from fifteen to forty minutes, and continued, without much variation, during 20 to 30 minutes. After this period, the whole effect recorded under this head diminished, and oftentimes suddenly, as was shown by the increased perception of light, as if a veil had fallen from the eyes, and by increased consciousness; but, nevertheless, the last power to be *completely* regained was consciousness.

(*c*) The increase in the action of the heart set in as soon as three minutes, and continued from thirty to fifty minutes.

(*d*) Coincident with this increase was a sense of dryness, heat, and evident fullness or swelling of the exposed parts of the skin, as the hands and face, and also a general sensation of heat. This was due to the increased supply of blood to the surface, and the lessened refrigeration of the skin. This increased for a time, and so much so, that, with rum especially, the skin was as harsh and dry as when exposed to an easterly wind. After about twenty to forty minutes, this sensation of heat gave place to one of cold, which was first felt on the most sensitive part of the body with regard to temperature, viz., between the shoulders, and at length, notwithstanding the existence of a suitable degree of atmospheric temperature, it became distressing, and led even to shivering. This was sometimes so marked, and occurred so suddenly, that it gave rise to a shock. It did not correspond with the temperature of the skin, but it was usually co-existent with the cessation of the increase of the heart's action.

(*e*) The muscular system was influenced in a marked and definite manner. The action on the involuntary muscular fibres of the heart has already been mentioned. The thin layers of voluntary muscles found about the body showed great relaxation. The respiratory muscles acted in a gasping manner, so that there was a pumping and quick respiratory effort in the later stage. At all periods there was a sense of impediment to respiration. The muscles of the limbs were inactive. There was relaxation of the muscles, and stiffness of the skin of the face, forehead, and upper lip, so that the features fell. This state of the muscular system followed the commencement of the effect upon the consciousness, and other functions of the brain, and also the excited state of the heart. In reference to its cessation, the power of co-ordinating the muscles was first regained, whilst the buzzing sensation and semi-cataleptic state continued, and the disposition to use the muscles was regained last of all. There was so close a connection between the nervous and the muscular systems, and complete consciousness is so essential in inquiries of this kind, that it is not easy to isolate the effect upon the muscles; but close attention to all the phenomena, the care to note them down at the moment of their occurrence, and the long series of experiments that we have made, assures us that the muscular tone and power are greatly lessened; that the effect is not identical upon voluntary and involuntary muscles, and that it is not even identical upon the inspiratory and expiratory set of muscles.

(*f*) The effect upon the mind is also very marked and peculiar, and would have been a very valuable study to a psychologist in search of facts. Rum and some other spirits made us very hilarious and talkative in about ten minutes, and during about twenty to twenty-five minutes; so much so, that my friend was almost a king; but as minutes flew away, so did our joyousness, and little by little we lessened our garrulity, and felt less happy, until at length, having gone down by degrees, we became silent, almost morose, and extremely miserable. Then, indeed, we felt the horrors and the sorrows of the drunkards lot, snd saw, with a clearness that can only be perceived by such experience, how certain it is that he must again drain the intoxicating cup. Never were the extremes of happiness and misery brought so vividly before us, or seemed to be in such close poximity, as on those occasions, and never did we so deeply commisserate the

6

slavish, miserable, and almost hopeless condition of the poor wretch who has become a victim to this fearful vice.

In addition to the above, we may mention that every mental perception was darkened, and that the dreaminess, which is not an unpleasant feature of it, is a condition in which neither thought nor imagination acquires power. We suspect very greatly the statements of those who profess that fancy is then on her most airy wing, or that thoughts spring forth without the efforts of parturition, and our most charitable reply to such statements would be, that it is all a dream.

(*g*) The effect upon the secretions was very marked, and, in addition to its varying effect upon the urine, it is certain that the secretion of the salivary glands and of the mucous membranes was lessened, as was shown by the dry state of the mouth, and by the sore and dry condition of the tip of the tongue, which was so often present when the rum was taken.

(*h*) The duration of the influence was varied somewhat, both with the substance taken, (being usually longer with rum, and shorter with gin,) and with the season of the year. In the spring time, it passed away within an hour and a half or two hours; but at other times the system continued to be disturbed, and we were depressed during the whole morning.

In a further inquiry which I have recently made in one of our prisons, I found that when a small quantity (half an ounce of alcohol) had been given four times a day to prisoners who labored at the treadwheel, it produced lassitude, thirst, and great diminution in the amount of urine. The prisoners remarked that they felt lazy and unfit to do their work, and slept more soundly at night.

Thus, the effect of alcohol, as shown by common observation, both of a general and precise kind, seems to show—

1. That the spirits may be excited, but all the functions of the brain are lessened or disturbed. The spirits are not the result solely of the action of the brain.

2. Muscular power is lessened.

3. The secretions of the salivary glands and mucous membrane, and, perhaps, of other organs are lessened.

4. The action of the skin is lessened.

5. The action of the heart is increased in force.

6. There is increased sensation of heat, due chiefly to the larger distribution of blood to the skin, (the organ of sensation of temperature,) and to the diminution of the loss of heat by diminished evaporation from the skin—a diminution not prevented by an increase in the loss of heat by radiation.

7. The sensation of cold in the reaction seems to be due quite as much to disturbed sensitiveness of the nervous system of the skin as to a withdrawal of the increase of the supply of blood to the skin, for it was far more severe than the external temperature warranted.

SCIENTIFIC RESEARCHES.

We may now turn to scientific research. The inquiries have been very numerous, but I will select Hammond as their type, because whilst his results have much corresponded with those of the other observers of his period, they are all liable to the same kinds of objection.

As to the influence of alcohols upon the respiration and the *heat-forming* function:

To chemists we owe the statement that alcohol aids in the heat-forming function of the body, because it is an hydro-carbon; and that it is transformed in the system, and eliminated as a carbonic acid and water, it must yield heat in these transformations. Hammond and all others found that alcohol lessened the quantity of carbonic acid expired, but no one has pointed out the inconsistency of this statement with the chemical view to which all these observers have given their adhesion—viz: that alcohol is resolved into carbonic acid, which must pass out by the lungs, and thereby increase the evolution of carbon. Lallemand, to whom we shall refer presently, when denying the transformation of the alcohol, says that he does not understand how alcohols lessen the excretion of carbonic acid, but upon this point he had not experimented.

Now see the objections which may be fairly raised to the experiments from which these results have been obtained:

1. Hammond collected the quantity for one minute three times a day, and thence inferred the total quantity in the twenty-four hours. This evil is also found in most other experiments, as those of Prout, Vierordt, Coathupe, Bocker, and others.

2. Hammond assumed that he ordinarily inspired 14 times a minute in health, and during all his experiments he did the same, and tried to inspire the same quantity as in health. Hence, at the most he could only have sought for the per centage of the carbonic acid in the expired air, and not the total quantity evolved in a given time; but the attempt to voluntarily limit the respiration deprives the whole process of value. Prout also sought only by the per centage amount. Further,

Hammond expired *through* a volume of solution of baryta, and thereby placed a weight upon his expiration, which would either prevent the completeness of the act when the respiratory power became feeble at the end of expiration, or it would demand an unusual force in expiration, which no man can so measure as to make the result correspond with free respiration.

3. The influence of other food was assumed to be the same from day to day, because the same amount of food was taken, and the same exertion made, and, moreover, it was supposed to be the same at the three particular minutes at which the carbonic acid was collected; but this is quite a fallacy, as we have shown in the hourly variations in the evolution of carboni acid. ("Philosophical Transactions," 1859.) The same admixture of other unknown influences from food, and the same want of knowledge (the knowledge did not then exist) of the natural variation in the amount of carbonic acid evolved at the various hours of the day, and of the variations from day to day, apply also to the experiments of Vierordt, Bocker, Coathupe, and other observers. Hence the liability to error in inferring large from very small quantities, and in assuming that conditions are constant whilst they are most variable, lessens vastly the reliability of the results obtained. To this might be added, in reference to Hammond's experiments, the imperfection of his apparatus; for it is quite certain that a tube of chloride of calcium would not absorb all the vapor from the breath during the act of breathing, and, also, that he ought to have appended a desiccator, through which the air should pass after the partially dried air had passed through the solution of baryta.

The results of my experiments have not entirely corresponded with those now referred to; but having collected the carbonic acid for five or ten minutes at a time every fifteen minutes during the action of the alcohol, with the expiration quite free; having maintained a uniform state of rest; having experimented in the absence of food or any other cause of disturbance, and having taken one moderate dose at each experiment, it is hoped that the nearest approach to the true action of alcohol has been made. Moreover, the number and extent of the experiments far exceed those which all previous observers have recorded. For the description of the method and apparatus employed, as well as the details of the experiments, I must refer to the "Philosophical Transactions" for 1859.

Alcohol, in every dose up to the usual one in taking spirits and water, increased the amount of carbonic acid evolved, but not with the uniformity of a food, nor to a greater average extent than one-eighth to half a grain per minute. The effect was much more sustained when a small dose, as half an ounce was repeated every quarter of an hour. Prout, and all observers, have noticed the want of precise uniformity in the action of alcohol.

Rum increased it somewhat more, and on some occasions much more. It is a spirit which is colored with burnt sugar, and we have proved that sugar very largely increases the evolutions of carbonic acid, but the cause of the increase has not been established.

Whiskey varied according to the sample.

Brandy and gin (both good, the former foreign and the latter unsweetened) lessened the quantity of carbonic acid evolved to a moderate extent generally, but sometimes gin caused great depression.

The duration of this action was from one to two hours.

The volatile oils and ethers which give character to the various wines and spirits, always, when inhaled, lessened the quantity of carbonic acid and vapor exhaled; and this was the greatest when the aromas were the best—viz: in old and fine wines and spirits. This result was without an exception. The effect of fusil oil, and other unknown compounds which cause headache, was not separately determined.

With all these substances there was variation in the effect, both in the same and different persons, and we characterized them as disturbers of the system.

Ales always increased the amount of carbonic acid evolved, to an average degree of a half to one grain per minute, and through a duration of two hours. They contain, besides alcohol, sugar, nitrogenous matter, and volatile aromas, and to the two former this effect must be attributed.

Rum and milk had a very similar, but greater and more sustained action.

Here I venture to affirm that alcohol, as a general stimulant, slightly increases the vital action of respiration; that gluten and sugar do so more largely; and that the aromas and probably the fusil oil and other injurious ingredients of bad spirit lessen these changes.

Now, in reference to the *flesh-forming* or the nitrogenous process. I do not stop to show that the term "flesh-forming" is derived from the supposed action of nitrogenous matter, as the waste of flesh is supposed to be indicated by the evolution of nitrogen; but it can be readily shown that urea may proceed either from the tissues, from the transformed food, and cannot therefore at present be regarded as indicating one or other separately.

Hammond showed that alcohol greatly lessened the excretion of urea, and in this he is supported by all observers. But mark the attendant circumstances, as showing the cause for this diminution. He says: "Whilst these experiments were progressing, the healthy action of my system was much disordered. Headache was constant, sleep was disturbed, the skin was hot, pulse full and bounding, averaging 98 per minute; and there was on two occasions after eating slight palpitation of the heart. My appetite was capricious; sometimes disgust was created by the mere sight of food, at other times I ate with a good deal of relish. I should think I should have been seriously ill if I had continued the investigation longer."

Hence it is quite certain that, whatever might have been the physiological effects of the alcohol, they were those of disease, and not of health; and that, although he ate from sixteen to twenty ounces of meat and other food proportionately daily, there was most marked disturbance of the assimilative function. With a large dictary he was ill-nourished, and hence, with lessened transformation of food there was a lessened amount of urea evolved. This is further proved by a reference to his statement that he gained weight (as confirming his view of the power of alcohols to retard waste,) for there was a gradual accumulation of fæces, as was shown by his statement: "This fortunate termination was probably promoted by a diarhœa, of considerable violence, which commenced on the second day after the conclusion of these experiments, and continued forty-eight hours." Moreover, I have proved that the system varies greatly in the amount of fluid which it appropriates, and that with alcohols taken daily, the amount of urine is lessened, and the system has acquired an increased power to accumulate food. This accumulation, like that of fæces, is, however, temporary, and, with elimination of it, all unpleasant symptoms pass away. In our experiments in prisons, we found that in all the four men the quantity of urea evolved was greatly lessened on the first and second day, but on the third day it was almost restored. During this period of retention, the body would gain weight, quite as great in extent as that proved by Hammond, but it would be no proof of diminished waste of tissue.

It may be properly objected, that the above mentioned statement of Hammond, as to the influence of alcohol upon his health, only refers to the two series of experiments in which he ate his ordinary amount, and an excessive amount of food, and that he found no such results to follow its employment when he ate a deficiency of food. This is true; but it does not affect the conclusion at which he arrives, viz., that the alcohol arrested waste of tissue, because, whilst the lost weight daily before he took the alcohol, he ceased to do so when alcohol was added to his diet, and at the same time the amount of urea was diminished. Let us for a moment see what value ought to be attached to this statement.

Before the experiment began, on an average of five days, his weight was 225-97 lbs., and the daily weights of the fæces and urine were 6 oz. and 41-29 oz. At the end of the experiment, on the average of five days, he had lost, as compared with the previous five days, 47 lbs. in weight, and the daily weight of his fæces and urine was lessened by -19 oz. and 1.37 oz. The whole question rests upon the fact of there being an arrest of the waste previously going on during the 5 days before the alcohol was taken, and a positive average gain of 0-3 lb. in the weight of a man weighing upwards of fifteen stone as compared with the weight in a single day immediately preceding the experiment. Is it not absurd to attach any value to so small a change, and particularly when we bear in mind the defects of ordinary balances to weigh 226 lbs., the errors of weighing, and the variation of excretion remaining within the body? But let us admit it all, and what is it worth? He gained in weight -16 lb. on the average of five days, whilst the retention of fluid per day in the urine during the five days was, on the average, -74 oz, and that of the fæces -25, or a total retention of excretions of 4-9 oz., from which he ought to have gained much more than he did gain. That these excretions were retained, and were subsequently emitted, is proved by the results of his own and of our experiments. Hence, he gained in weight by the temporary retention of urine and excretion, and not by any increase in the tissue of the body, and the whole supposed value of the experiment and the deductions therefrom is taken away.

Hammond further states, tbat the perspiration did not appear to be lessened under a temperature of 73° in the shade; but he did not make any experiments in reference to it, and referred only to the evident perspiration. Other experimenters, both upon men and animals, have found the skin unusually dry and harsh.

To these various scientific researches, we will add those of MM. Lallemand, Perrin and Duroy, who have just shown that when a moderate dose of alcohol has been taken, either by a man or dog, the alcohol can be found in the expiration, the perspiration, and the urine for at least eight hours afterwards. They have not collected the alcohol, but they have shown its presence by its action upon the bichromate of potass in sulphuric acid, and have proved that this action is not produced by the allied substance aldehyde. They affirm, therefore, that alcohol is not transformed in the system, but passes off the body unchanged; and the fact that alcohol is found in the brain for a period of seventeen, and it said for thirty-six hours, strengthens their belief. I have carefully repeated their experiments, and have shown on several public occasions, the reaction of alcohol on the breath for four hours after an ounce and a half has been taken. In the case of the skin, M. Lallemand proved the secretion of alcohol, by enclosing an Italian greyhound in a glass case, and passing a current of air over it and through the test; but I have proved its presence in the vapor passing off from so small a part of the body as the arm, and even the hand only, of man. In repeating this latter experiment, sheet caoutchouc must not be used, neither must the containing bag or vessels be employed a second time without having been well cleansed from the alcohol which was left from the former experiment. It will also be more efficiently performed in those persons who perspire freely; for it is highly probable, that whilst alcohol may in all persons pass out of the body by all the above mentioned outlets, it will chiefly select that excretion in each individual which is most free. As, for example, the breath in those who make much exertion, or in cold; the skin in those who perspire freely, and in warm weather; and the kidneys in others. The test is prepared by carefully dissolving one part of bichromate of potash in 300 parts of strong pure sulphuric acid, and it is used by passing the breath through absut a drachm of it placed at the bottom of a tube six or eight inches long. The cherry red color is changed to emerald green.

Thus we are led to the following summary of the mode of action of alcohol:

1st. The action of the heart is reinforced, and by it the fullness and freeness of the circulation are maintained, and the blood carried to the remote parts and to the surface. The tendency to accumulate fluid increases the fullness of the bloodvessels.

2d. The action of the skin is lessened, whereby the loss of heat is reduced, the urgent necessity for food and vital transformation is lessened, and the sensation of warmth is increased. The local action upon the stomach and alimentary canal is that of a stimulant, and increases vascular action and warmth. These are the common and general actions of alcohols; but in some persons the skin acts more freely than in others.

3d. Alcohol is probably not transformed, and does not increase the production of heat by its own chemical action, but indirectly, as above mentioned, and by a general temporary increase in the vital actions. Its action upon the respiration is not its important action.

4th. It interferes with alimentation, and causes the retention of water, and thereby lessens the excretion of urea; and if it lessens muscular waste (which it has not been proved to do), it must be by lessening vital action. Its power to lessen salivary secretion must impede the due digestion of starch. The diminution in the elimination is due in part, as just mentioned, to the excretion of urine by the kidneys.

5th. It lessens muscular power, and the production of certain secretions.

6th. There is no evidence that it increases nervous influence, except the action of the heart and elevation of the spirits be regarded as such; whilst there is much evidence that it lessens the nervous power, as shown by the mind and the muscles.

7th. It varies the balance of the circulation at the centres and the superfices, and interferes with the production of heat.

8th. It has two sets of actions.

9th. The system acquires an adapting power more or less easily and perfectly, and hence alcohols differ sometimes in their action in different persons.

10th. There are other important elements in ales, wines and spirits, having a different and independent action; so that old wines directly lessen vital action, and ales directly promote transformation of food.

The dose influences the phenomena of the action of alcohol, but only in degree, although it is common to consider the action to differ in doses which are called moderate and excessive respectively, the former being stimulating, and the latter exhausting. So far as the direct action upon the mind with its intellectual and sensual perceptions, upon the muscles, and upon the respiration is concerned, we could not see any difference in effect but one of degree; and, doubtless, in indirect effect, that dose which most powerfully acts upon the system directly will be the most likely, by the disturbance of the functions of the system, to produce indirect effects which will tend towards disease.

For all medicinal and dietetic purposes, I venture to affirm that the dose only affects the degree, and not the direction of the influence.

Having thus established the foregoing facts, I proceed to consider the views of the late Dr. Todd on the therapeutic action of alcohols, as contained in his well known lecture on that subject, and to see how far they may be supported by the present state of science. His views are based upon the chemical theory that alcohol is transformed within the system, and produces heat, and the following is a summary of his remarks:

1. Alcohol acts primarily on the nervous system, and, like other hydro-carbons, but in a greater degree, has great affinity for the nervous system.

2. It acts in two degrees: one beneficially, when it augments the generation of nervous power; and the other injuriously, by deteriorating, impairing or destroying the nutrition of nerve matter. In the first degree, and when it acts beneficially, there is no smell of alcohol in the breath; but in the latter or when the dose is too large, the odor is perceptible.

3. There is no true secondary depression of the vital powers, except when the quantity is too large, and then it acts by deranging the digestive functions.

4. Alcohol does not, in any dose, produce inflammation of the lungs, heart or liver. The brain symptoms do not show congestion or inflammation, but a poisoning of the nerve-cell and nerve-fibre.

5. When carefully taken, it upholds the calorifying process, strengthens the action of the heart, and reduces the frequency of the pulse. By upholding the calorifacient process, it prevents oxydation of the nervous and other tissues.

6. It is simply absorbed into the circulatory system; but oil (another hydro-carbon) has a more complicated digestion, and therefore alcohol acts more quickly and certainly.

7. The dose should be from two drachms to two ounces, and repeated as frequently as food would be given, and with the same intention—viz, to save tissues from oxydation. The dose and the frequency should be adhered to.

8. The general use of alcohols shows an instinct in man for them, and in recovery from disease they uphold nervous force and supply the most assimilable material for com bustion. They calm the nervous system and avert delirium

9. It is very dangerous to withdraw the alcohol, but the signs which would sanction this are referable to deranged digestion—as flatulus, eructations, sickness, and dry tongue and mouth; and also the smell of the breath which implies that the alcohol is passing off unchanged. Coma and delirium from excess of alcohol rather indicate that more alcohol should be given.

Such is a summary of Dr. Todd's views, and they chiefly include the belief that alcohol increases nerve-nutrition, strengthens the heart's action, lessens the rapidity of the circulation, shields the tissues from oxydation, and produces heat; and the sign which measures the efficiency of it is the non-existence of the odor in the breath. As to the skin, he merely remarks that when alcohol is given to a patient who has a hot skin and a rapid pulse, it may look like an illustration of the dogma, "Similia similibus curantur." In reference to all his inferences from the supposed transformation of alcohol in the system, we may remark that they are of no value if it be shown that this transformation does not occur, but that alcohol, after remaining in the system for a time, and disturbing its action, is ejected still as alcohol. I believe that the latter is almost if not quite certain.

I see no ground whatever for the statement that hydrocarbons have an especial affinity for the nervous system, taking starch and oils as the representative of this class, and Dr. Todd does not adduce any. Neither has it been in any way proved that alcohols improve the nutrition of the nerve cell and fibre in any separate and special way, but only by improving the whole nutrition of the body. Indeed, the phenomena of nutrition lead our attention away from mere local actions as independent conditions, and teach us that it is a general and not a local act. All the phenomena attending upon alcohols when taken in quantities which may render their effects noticeable, evince great nervous disturbance; and I cannot but believe that, whilst the nervous system is principally affected, it is by the opposite of a nutrient and healthy action.

The statement as to the action of the heart is doubtless correct, as is also that upon the frequency of the pulse; but the latter action is indirect, and probably due to the former, whereby a larger quantity of blood is forwarded at each contraction of the heart, and the blood is more perfectly distributed to the whole body. A rapid pulse is commonly a small one, and the impulsion of a large quantity of blood at a time lessens the necessity for the quicker circulation of it.

It is singular that so acute an observer should have made so little reference to the effect of alcohol upon the skin, its power to accumulate water in the body, and to retard the elimination of urea; but in reference to the former I may remark that acetate of ammonia was a most frequent adjunct to his alcoholic treatment, and this might do much to counteract the action of alcohol upon the skin. Moreover, whilst he did not withhold alcohol when the skin was hot and dry, his cases were frequently such as had perspiring skins, and in such instances he remarked upon the diminution in the perspiration during the action of alcohols. It may also here be observed that Dr. Todd most commonly added chloric ether, and not unfrequently quinine, to his plan of treatment, and hence there were several actions induced, some co-ordinate and others opposed.

His statement in reference to the absence of alcoholic exhalation from the lungs in cases of diseases in which the alcohol is well borne, is worthy of further investigation; but on inquiry I do not find that this fact has been recognized by others. If alcohol passes off untransformed, it is evident that this cannot be true; but if it be true, it is a most valuable guide.

Upon the whole, I do not think that the arguments used by Dr. Todd are now sufficient to establish the theory of the action of alcohols, or to warrant his peculiar plan of administering them; but, on the other hand, I think that the practice which he pursued must rest only upon the ground of his personal authority.

It now remains to state the conditions and stages of disease to which alcohols, in various forms, seem to be fitted—the action in health being accepted as a sufficient guide—and it will not be difficult to show that the usual practice of the profession has been based upon truth, however little or much the mode of action may have been understood.

I think that it must be accepted that *the essential and direct actions of alcohols, as remedial agents, are, the increase of the force of the heart's action, the local stimulation of the stomach, and the diminutian in the action of the skin;* whilst the most important independent actions are, the retention of urea and fæces by or with retention of fluid, diminution of certain secretions, and derangement of the assimilative process.

In conditions in which the force of the circulation is too feeble, there must be defective nutrition, innervation, and oxydization of the products of alimentation; for a certain fullness of the bloodvessels is clearly necessary to such brain phenomena as consciousness, as may be inferred from fainting; to excretion, as may be seen by the increase of urine after ingestion of fluids; to general innervation, by the sense of *malaise* which accompanies feeble and of *bienaise* which is found with proper heart's action; and a certain frequency and duration of exposure of the blood in respiration, is necessary to the proper interchange of gases, whilst a due amount of action of the heart, as well as of inspiratory effort, is required for the free circulation of the blood through the lungs. Hence, with weakening of the force of the heart, the muscular and nervous forces are weakened, and all vital processes are inadequate to health.

Such a state is that of general debility, deficient innervation, or exhaustion from numerous causes, temporary debility from over-exertion or anxiety, convalescence from almost all diseases, and the state of exhaustion in fevers with certain limitations.

If, in such conditions, alcohol be administered in such a manner as not to induce derangement of the system, it is easy to see how Dr. Todd's statement may be verified—viz., that it improves the nutrition of the nerve-structures, and does not produce inflammation; and, in addition, it may be shown that, by its power to increase the force of the circulation, it will prevent or remove the tendency to local congestion of organs, as the lungs and liver, and the results of such congestion —viz., effusion of fluid—and will improve the whole nutritive and vital powers of the body. This is certainly in accord with universal practice, both in this and former ages, for whilst we may appeal to the great mass of the profession in reference to their experience now, we have on record, in the works of Artæus, an admirable description of the effects of wine in such conditions in his day, which we will transcribe. In the treatment of syncope, he remarks: "But if converted into syncope, and this also happens, (the power of life being loosened, the patient being melted in sweat, and all his humors being determined outwardly, the strength and pneuma being also dissolved,) we must disregard the delirium, and be upon our guard lest the patient be resolved into vapor and humidity.—Then the only support is wine, to nourish quickly by its substance, and to penetrate everywhere, even to the extremities, to add tone to tone; to rouse the torpid pneuma, warm that which is cold, brace what is relaxed, restrain those portions which are flowing outwards—wine being sweet to the sense of smell, so as to impart pleasure; powerful to confirm the strength for life, and most excellent to soothe the mind in delirium. Wine, when drunk, accomplishes all these good purpose', for they become composed by the soothing of their minds, are spontaneously nourished to strength, and are inspired with pleasure."

But we require at least one other indication as our guide in the administration of alcohol, viz., the condition of the skin. The regulation of the heat of the body, as well as the due distribution of the volume of the blood to the central and perpheral organs, rests very much with the skin. We know very well the great benefit which results in the last stage of fevers in local inflammation, and in sun-stroke, from the free use of cold water to the skin, although that cools the body only by contact and radiation; and of the application of warmer fluids in similar conditions, which, so far as they were of lower temperature than the skin, directly cooled the body, and so far as they approached or exceeded that of the skin, and thus indirectly cool the body. Both these plans are of well known efficacy, and although apparently opposed, produce, or tend to produce, by different methods, the one result. Now, although there may be a deficiency in the heart's action, if the skin be hot and dry, it is manifest that great risk may follow the use of alcohols; and when, in the sthenic stages of fevers and inflammation, the force of the circulation is increased, from the resistance to the current of blood offered by the capillaries in the great inactive external organ of the body—the skin—it must tend directly to do injury. Hence, it was never the practice to give these substances in the active and hot stages of fever; but when the condition arrived in which the skin lost something of its dryness, and became soft, and particularly when it perspired, alcohols and bark became the sheet-anchor of the practitioner. With a perspiring skin, wine and other alcohols could be given profusely, and I once gave six bottles of port wine to a female in forty-eight hours, in such a condition occurring in fever. These are the states in cholera and in diphtheria in which very large quantities have been administered, with the effect of saving life from imminent peril.

Such, also, were the views of the ancients, for Arctæus again writes: "But if the period be already come to a crisis, if there be dew on the clavicle and forehead, the extremities cold, the pulse very small and frequent, or if creeping and feeble in tone, the patient must take a little food and partake of wine effectually." And again: "But if much sweat flow, the pulse come to a stop, the voice become sharp, the breast lose its heat, we are to give as much wine as the patient can drink. For those who are cold, wine is the only hope of life."

Hence, a feeble pulse and an active skin are, and have always been regarded as the clear indications of the requirement of alcohol, and as the skin loses its exces of action, the rapidity of the pulse will decline, and its fullness and force increase. This is most perfectly exemplified in the case of many Europeans, even ladies, living in India, who find it really impossible to sustain innervation without the use of some forms of alcohol.

I have before referred to Dr. Todd's practice of giving acetate of ammonia, and may remark, in cases of fever, when the exhaustion had just begun, it was always the rule among practitioners to give ammonia, and not an acid, with the cinchona, with or without wine; and no doubt the true action of this was to temper the action of the bark and the alcohol upon the skin, so as to prevent a too rapid reduction of the action of the latter organ. It is, however, singular, that patients with hot and dry skin should have borne the exhibition of twelve or twenty ounces, and even much more of brandy daily, for weeks together, whatever may be the truth as to mortality or prolonged duration of the disease which followed its use. We see in health that there is a remarkable variation in the toleration of the system to the action of alcohol, and in certain conditions of disease we know that the toleration is much greater. If Dr. Todd's statements be correct as to the non-emission of the fumes of alcohol by the breath of certain cases in which large quantities have been given, it is quite open to question if, in such cases, the alcohol obtained its usual entrance into the circulation. This seems to be the explanation of the similar condition in cholera, where the vital power of absorption is greatly reduced, and it is quite certain that the body has naturally a power to limit the introduction of food and water into the blood, but allows it to pass off by the bowels. This is, probably, the safety-valve provided for such emergencies.

It has fallen to my lot to see cases of subacute inflammation of the lungs, and of the deposit which has succeeded to the inflammation, treated on Dr. Todd's method with alcohol, whilst the action of the skin was deficient, and I certainly have been impressed with the belief that the progress of such cases towards recovery was greatly retarded.

The action of alcohol in restraining the secretion of the saliva and of the mucous membranes did not appear to me, in such acute or subacute cases, to be of great importance; for whatever might be the state of the tongue, if the skin were freely acting, and other conditions called for the use of alcohol, the alcohol was well borne.

It is impossible to pass over in silence the influence of alcohol in lessening the excretion of urea; for if it do so simply by causing the retention of fluid in the body, it may be most prejudicial in cases of fever, where the whole safety of the patient seems to depend upon the elimination of the excessive amount of urea which then occurs. It is only when the amount of urea has naturally fallen with the subsidence of the febrile condition that alcohol can be safely administered.

But in any condition in which the amount of urea is lessened by reason of interference with the digestion and transformation of food, it is allowed by all that alcohol is injurious. In this condition it is very likely that the diminution in the flow of saliva may play a very important part, for it must greatly lessen the transformation of starch, and the retention of the fæces with other co-ordinate actions will explain the temporary condition of the liver and the headache, which are their constant attendants.

The efficacy of alcohol in delirium and coma has been quoted as a prime argument in support of recent views.—When the delirium is not the result of blood and brain disease, but indicates exhaustion, as in delirium tremens, syncope, or after fright, it is easy to explain that alcohol, opium, or bark, by giving force to the heart, and lessening the action of the skin and kidneys, is very likely to cause that condition of fullness of the bloodvessels of the brain which is necessary to perfect consciousness. In certain forms of coma the beneficial action of alcohol may be explained by its power to quicken the circulation. In both of these states we shall doubtless find that any remedy which acts in the manner now indicated will be equally beneficial with alcohol. Whilst there is so much diversity and apparent opposition in the remedies employed by various practitioners, there can be no doubt that they have a unity in the mode of their action which it is most desirable to discover.

The local action of alcohols upon the stomach does not call for remark since the direction of their action is evident and well understood. The statement that alcohol must impede digestion, because when a substance is placed in it it becomes hard, can have no reference to the action of the moderate and diluted doses or alcohols which are usually employed in medicine.

The frequency and regularity with which alcohol should be given, when it is necessary to prevent exhaustion, have been well stated by Dr. Todd. As the primary action is temporary and passes away in half an hour, the call for it is quickly renewed, and in cases of imminent danger an interval of half an hour would be long enough.

As to the dose, I venture to recommend the use of small doses—say half an ounce of alcohol where spirits would be given; for we have shown that the frequent repetition of a small dose conduces both to a full and sustained action. It may be well to remember that, as spirits contain from 40 to 70 per cent. of alcohol, sherry and port wines from 17 to 24 per cent., and good ales from 6 to 10 per cent., two ounces of spirit are equal in alcohol to about six or eight ounces of wine, and twelve to sixteen ounces of ale.

In conclusion, I would invite attention to the use of the aromas of wines and the saccharine and nitrogenous elements of ales. It is clear that these have an action apart, and sometimes different from the alcohol, and therefore are fitted for different conditions. The conservative influence of the aromas of wines seems especially to be fitted for the cases of excessive nervous action, in which to reduce the action would conduce to health; whilst in fever it has been remarked that the newer wines, containing little aroma and more spirit than old wines, have a better action.

It is well known that a quantity of alcohol may be taken in wine, and particularly in good wine, in health, without affecting the sensorium, which could not be borne at all if drunk as spirits and water—a fact due partly to the greater length of time over which the drinking of wine is extended, and in other part to the opposing effect of the aromas. The value of the aromas has been recognised in all ages, and Artæus directs that "the wine is to be fragrant and not very astringent."

As sugar largely increases the evolution of carbonic acid, and gluton has a similar but less and less rapid action, and in the latter case at least the carbon could not have proceeded from the gluton, I am led to believe that beers have the power of directly promoting the assimilation of carbonaceous food. Whether this is due to the quality of a ferment which nitrogenous matters possess, it is not here important to discuss; but the combination of alcohol with these principles, and with a bitter property, gives the most admirable compound which the word has seen, and especially fitted for the cases in which there are general enervation, defective powers of assimilation, and active state of the skin, as in the lassitude and exhaustion of many in hot climates.

Hence, there are certain cases in which alcohol is chiefly called for, and in which spirits of wine may be given quite as usefully as the ordinary compounds; others in which the aromas of fine old wines are of great value; and a third class in which the addition of the nitrogenous digestive agent in ales should be given.

I think it would be to the advantage of our patients if, in cases in which we desire the effect of alcohol, and not the other ingredients found in alcohols—viz., increase in the action of the heart and decrease in the action of the skin—we administered spirits of wine instead of spirits, because by obtaining it during the first hours of distillation, we may obtain nearly a pure alcohol, and by indicating the specific gravity we may at all times know the true amount of the agent which we would employ. Although alcohol varies very greatly in flavor and degrees of purity, and it commonly happens that the kind which is used in the manufacture of spirits is that which passes off in the latter hours of distillation, and contains much fusil oil and free acid, which are very injurious to health. Moreover, as there is a clear difference in the action of alcohol and fine old brandy, we have in alcohol an agent which will effect the two purposes above mentioned, and will at the same time support the respiratory organs.—*London Lancet.*

